# A phenomenological study of new doctors’ transition to practice, utilising participant-voiced poetry

**DOI:** 10.1007/s10459-021-10046-x

**Published:** 2021-04-13

**Authors:** Megan E. L. Brown, Amy Proudfoot, Nabilah Y. Mayat, Gabrielle M. Finn

**Affiliations:** 1 Health Professions Education Unit, Hull York Medical School, York, UK; 2 Barts and the London School of Medicine and Dentistry, London, UK; 3 School of Medical Sciences, Faculty of Biology, Medicine and Health, The University of Manchester, Manchester, UK

**Keywords:** Ethic of caring, Foundation programme, Moral injury, Participant-voiced poetry, Phenomenology, Poetic Inquiry, Transition, Transition to practice

## Abstract

Transition to practice can be a turbulent time for new doctors. It has been proposed transition is experienced non-linearly in physical, psychological, cultural and social domains. What is less well known, however, is whether transition within these domains can contribute to the experience of moral injury in new doctors. Further, the lived experience of doctors as they transition to practice is underexplored. Given this, we asked; how do newly qualified doctors experience transition from medical school to practice? One-to-one phenomenological interviews with 7 recently qualified UK doctors were undertaken. Findings were analysed using Ajjawi and Higgs’ framework of hermeneutic analysis. Following identification of secondary concepts, participant-voiced research poems were crafted by the research team, re-displaying participant words chronologically to convey meaning and deepen analysis. 4 themes were identified: (1) The nature of transition to practice; (2) The influence of community; (3) The influence of personal beliefs and values; and (4) The impact of unrealistic undergraduate experience. Transition to practice was viewed mostly negatively, with interpersonal support difficult to access given the 4-month nature of rotations. Participants describe relying on strong personal beliefs and values, often rooted in an ‘ethic of caring’ to cope. Yet, in the fraught landscape of the NHS, an ethic of caring can also prove troublesome and predispose to moral injury as trainees work within a fragmented system misaligned with personal values. The disjointed nature of postgraduate training requires review, with focus on individual resilience redirected to tackle systemic health-service issues.

## Introduction

If tomorrow we all awoke gifted with the talents of a certain Dr Dolittle, it would be interesting to know a butterfly’s experience of metamorphosis. Although now perceived by us all as a spectacularly transformed red admiral, monarch or cabbage white (types of butterflies for those not entomologically inclined), just what is happening behind-the-scenes of their transformation? What was it like for those fourteen to seventeen days that caterpillars spend pupating? What is it like for those butterflies now, as blood is pumped to their wings and they learn to fly? These are the questions we should also ask of our new doctors, as they undergo significant, although likely not entirely metamorphotic, professional change. Like metamorphosis, this professional change- encapsulated within medical education research by the more nuanced term ‘transition’- is complex. Transition has been defined as ‘an ongoing process of psychological, social and educational adaptation over time due to changes in context, interpersonal relationships and identity’ (Gordon, Teunissen, et al., 2020; Jindal-Snape, 2018). Beginning work as a newly qualified doctor represents a transition in role and responsibility for medical graduates. Upon qualification, a new junior doctor flies forth into the world of medicine and must adapt to their new life with wings. Preparing students for this transition has traditionally been the focus of research in this realm (Illing et al., 2013; Monrouxe et al., 2014; Monrouxe, Grundy, et al., 2017; Tallentire et al., 2011), with previous work revealing a large discrepancy between student experience and qualified practice which negatively impacts patient care (Barnes et al., 2019; Morrow et al., 2012; Wells et al., 2019). Transitions within medical education are increasingly the focus of research, but most work focuses on either undergraduate or higher-stage postgraduate trainees (Gordon et al., 2017; Gordon, Rees, et al., 2020), or approaches transition through specific lenses, such as clinical competency (Lempp et al., 2005), or professional identity (de Lasson et al., 2016). There is a relative dearth of research regarding the *lived experience* of new doctors’ practice during transition to their first postgraduate year.

Beginning work as a doctor has been proposed by Teunissen and Westerman (2011b) as one of three major transitions within medical education (the other two transitions being the shift from non-clinical to clinical training within medical school, and specialist trainees’ transitions to medical specialists later within doctors’ careers). The approaches to conceptualising the transition which concerns beginning work as a doctor have been broad. In their recent scoping review Atherley et al. (2019) note that transition can be conceptualised from three distinct perspectives: educational, social and developmental. Educational perspectives towards transitions focus on “trying to narrow the gap between [transitions]”; social perspectives focus on “address[ing]… struggles when transitioning… by focusing on relationships”; and developmental perspectives conceptualise transitions as influencing personal and professional growth. Although Atherley et al.’s classification was created through analysis of undergraduate literature, there are parallels with research conceptualising transition to practice. The bulk of available research conceptualises this transition educationally, focusing on preparing students for practice and, subsequently, new doctor performance and ability (Monrouxe et al., 2018; Monrouxe, Grundy, et al., 2017; Teunissen & Westerman, 2011a). Social perspectives are adopted less frequently, although transition has been explored through sociocultural lenses (Gordon et al., 2017). Contemporarily, conceptualisation of transition as developmental has been most populous, with its impact researched through the lens of professional development, including processes of identity formation (Rees, 2017; Yu et al., 2020), the emotional impact of transition (Fenwick et al., 2013), and methods of emotional regulation (Lundin et al., 2018).

In line with a developmental perspective, Multiple and Multidimensional Transitions Theory (MMT) was proposed by Jindal-Snape et al. in 2016 and has been subsequently applied to new doctors’ transitions to practice (Gordon et al., 2017; Gordon, Teunissen, et al., 2020). MMT conceptualises transitions as non-linear processes and maintains that “individuals inhabit multiple domains…. [which] can be physical, cultural, psychological and social”. Within MMT, each of the domains an individual inhabits plays an important role in transition- they are interconnected, a change in one domain can trigger change within another- like a cocoon, transition is a layered phenomenon, involving ongoing interaction between multiple territories (Gordon et al., 2017). However, there are gaps in what is known concerning how these domains interact and impact trainee experience during transition. In particular, the influence of transition on new doctors’ experiences of phenomena such as moral injury, which has cultural, psychological and social domains, remains unclear. Moral injury occurs when “there has been a betrayal of what’s right, by someone who holds legitimate authority, in a high-stakes situation” (Shay, 2002), and is acclaimed by some as the cause of medicine’s burnout epidemic (Mehta & Mehta, 2019). Murray et al. (2018) have demonstrated the utility of the term moral injury in exploring the experience of undergraduate medical students within emergency medicine, yet, investigation of moral injury amongst new doctors is sparse, with most published work taking the form of commentaries (Dzeng & Wachter, 2019; Howe et al., 2012; Talbot & Dean, 2020). The evidence for burnout during transition, speculated by some to represent the ‘end-stage’ of moral injury, is more significant. Monrouxe, Bullock, et al. (2017) report that the incidence of burnout increases throughout new doctors’ first year of practice, whilst Hariharan and Griffin (2019) identify poor workplace culture, unsatisfactory experiences of supervisory relationships, and high workload as factors associated with burnout in early-career doctors. Whether moral injury plays a role in such burnout of new doctors remains unclear, as empirical investigation of new doctors’ experiences which may contribute to moral injury is lacking. Fundamentally, moral injury is thought to follow a mismatch between what an individual holds to be just and the world they must participate within (Murray et al., 2018). Although discrepancies between new doctor’s initial perceptions of their role and their role in actuality have been previously reported (Vance et al., 2019; Yardley et al., 2020), it remains unclear whether these discrepancies cumulate in a perceived difference between a practitioner’s practice and their ideals that then disposes to moral injury. Further to this, the relationship of moral injury to the domains of MMT theory has not yet been investigated.

As such, scrutiny of new doctors’ experiences as they begin practice holds merit. Although we have analysed new doctors’ performance and targeted this beautiful display of skill through the lens of ‘preparation’, we have much less frequently focused on the more complex nature of *experiencing* transition to practice. Of particular note, exploration of new doctors’ experience from a phenomenological standpoint is lacking. As most prior research has been conducted through particular lenses such as professional identity development, thick descriptions and analyses of transition to practice as an experience are wanting. Whilst this research aims to address gaps within transition to practice literature (namely the experience of moral injury during transition), other, unidentified influences associated with new doctors’ transition to practice may be identified through exploration of participants’ lived experience. Given the need for phenomenological conceptualisation of transition to practice, therefore, we asked: how do newly qualified doctors experience transition from medical school to practice within the UK foundation programme?

## Methods

### Methodological orientation

Hermeneutic phenomenology was selected to assist in painting a vivid picture of participant lived experience regarding transition. Hermeneutic or interpretative phenomenology explores phenomena as they exist in the consciousness of the perceiver (Smith et al., 1999), placing value on the subjective nature of participant understanding (Eatough & Smith, 2008). Hermeneutic phenomenology moves beyond mere description of events, into exploring “the meaning of lived experience, and the contextual forces that shape it” (Bynum et al., 2019). Operating within an interpretivist paradigm, we acknowledge reality and knowledge to be subjectively constructed by individuals (Young & Collin, 2004). Given this understanding, hermeneutic phenomenology, that pays heed to the subjective nature of reality and emphasises context, seemed an appropriate choice to investigate individual’s transition to practice.

Hermeneutic phenomenology recognises the impossibility of ‘bracketing off’ personal experiences and becoming a neutral research party (Eatough & Smith, 2008). Instead, prior assumptions are reflected upon, made clear, and embraced in the process of study design. Two researchers are qualified doctors (MB and AP) and relatively recently experienced transition to medical practice themselves (4 years ago). MB is currently non-practicing, pursing a full-time PhD in medical education, and AP works as a general practice registrar. NM is a medical student in her penultimate year study, who has previously researched the transition from the pre-clinical to clinical years of medical education. GF is a professor of medical education, with interests including professionalism and professional identity formation. Reflexivity discussions were held throughout, increasing the authors’ awareness of their own views in regard to transition. NM views transition to practice as a large step, through the lens of a student yet to bridge this gap. Viewing transition as an unsurmounted hurdle may have coloured her interpretation. During their own recent transitions to practice, MB and AP both experienced periods of hardship, and, at times, support they perceived as insufficient, particularly when moving wards. Given this, MB and AP may have been more likely to identify negative experiences within participant interviews. GF’s interests in professionalism and identity formation likely influenced her interpretation, as she initially approached transition through a more specific ‘developmental’ lens than the phenomenological conceptualisation of this study. All authors kept reflexive journals to challenge their interpretations of participant accounts, reflecting consciously on these areas of their own experience that may influence interpretation. Through continual reflexive engagement, the research team’s prior assumptions allowed for a more considered analysis and increased empathic engagement with participants.

### Context

Within the United Kingdom (UK), the first two years of postgraduate practice take place within a national training scheme, the foundation programme. Medical graduates work as foundation year one and two doctors in turn, rotating through a pre-set list of medical, surgical and community jobs every 4 months (UK Foundation Programe Office (UKFPO)., 2019). Trainees are allocated one educational supervisor annually, although their on-the-ground clinical supervision changes as they rotate. Training occurs within the National Health Service (NHS), a public healthcare system, where care is free at the point of delivery. Training for new doctors was the subject of much debate over a decade ago with the foundation programme revised in light of this discourse in 2016 (Brennan et al., 2010; Soltan & Powell, 2016). The focus of this reform was upon “seamless transition” between stages of training (Peterkin and Bleakley, 2017), with independent inquiries highlighting the negative impact of turbulent transitions upon trainees (Monrouxe et al., 2014). Although the foundation programme only consists of two years, colloquially, doctors choosing not to directly enter speciality training are often referred to as ‘foundation year three’, ‘foundation year four’ etc. and ad infinitum, until they enter training.

### Data collection

Ethical approval was obtained from the Hull York Medical School institutional review board (approval number:1840). Audio recorded, in depth semi-structured interviews were undertaken with all participants, then transcribed verbatim by MB and AP. Participation was voluntary, not incentivised and written informed consent obtained. Semi-structured question stems were designed to prompt detailed discussion of the lived experience of becoming a doctor. All participants were led through debriefing material following their interview, where avenues of further psychological support were signposted to, if necessary.

In 2019 seven recently qualified doctors, all practicing in the UK, were recruited through advertisements on social media (Twitter and Facebook). As hermeneutic phenomenology recommends a small, homogenous sample (Willig, 2009), doctors were purposively recruited with recent experience of the transition to medical practice that occurs within the first sixth months of the foundation programme. Six months was selected as the transition time period for examination in this work, as previous phenomenological research regarding transition to practice in adjacent occupational fields have established 6 months as generating enough experience to evaluate this transition (O'Shea & Kelly, 2007). We defined ‘recent’ experience of these first 6 months of transition in this context as within two years of completing the foundation programme. Participant demographics are listed in Table 1.

**Table 1:** Participant demographic data

Demographic	Data
Age	Range: 24–29
	Mean age: 26
Gender	M:F 2:5

Training grade	Training grade	Number of participants
	Foundation Year 1	3
	Foundation Year 2	2
	‘Foundation Year 3’	1
	‘Foundation Year 4’	1

### Data analysis: hermeneutic phenomenological analysis and poetic inquiry

To lend structure to our analysis, Ajjawi and Higgs’ 6 stages of hermeneutic analysis (2007) were used as an analytical framework. Ajjawi and Higgs’ approach advocates initially for “identification of participants’ interpretations” or first-order constructs, which are then “layered with…researchers’ own understandings [and] interpretations” to generate second-order constructs (Ajjawi & Higgs, 2007; Bynum et al., 2019). The 6 stages of Ajjawi and Higgs’ analysis are: 1. Immersion; 2. Understanding; 3. Abstraction; 4. Synthesis; 5. Illumination; and 6. Integration. Within immersion, researchers engage themselves in the data they have collected, using techniques such as repeated reading, which fosters familiarity and facilitates coding. The second analytic stage, ‘understanding’, involves coding to identify first-order constructs. Within this research, first-order constructs were identified which expressed participants’ ideas and opinions in their own words. Stage three, ‘abstraction’, is the stage within which researchers’ own understandings are applied to the previously identified first-order constructs. The research team drew on their personal experiences, and theoretical knowledge of transition, to re-evaluate first-order constructs and abstract to second-order constructs. Within stage four, ‘synthesis’, themes are developed from second-order constructs. The research team cycled between theory, raw data, and earlier stages of analysis to identify sub-themes and themes. Cycles of writing were undertaken to elaborate on connections within the data. Stage five, ‘illumination’ involves “illuminating and illustrating the phenomena” (Ajjawi & Higgs, 2007)- in this case, transition to practice. Results were presented as a narrative to conceptualise the experience. The final stage, ‘integration’, concerns the testing and refining of themes. The research team engaged in detailed discussion regarding the narrative of results, evaluating the narrative alongside initial themes, second-order and first-order constructs for fit.

Alongside utilising Ajjawi and Higgs’ framework, the research team engaged in ‘methodological borrowing’ (Varpio et al., 2015), using a form of poetic inquiry known as participant-voiced poetry to deepen data analysis. In keeping with hermeneutic traditions, participant-voiced poetry facilitated data immersion and granted insight into the multiple ways in which participants experience and express the phenomena of transition to practice. Further, the use of participant-voiced poetry helped capture the complexity of transition, whilst respecting the differences between participant accounts.

Participant-voiced poetry is a form of poetic inquiry “where the researcher uses only the words of the participants gathered during research and re-presents them” (Gair & Van Luyn, 2016). It is similar to the practice of ‘found poetry’ (Sjollema et al., 2012), and is known by several names, including: data poetry (Sullivan et al., 2002); and vox-participare (Prendergast, 2009). The use of participant-voiced poetry is not new within qualitative inquiry (Butler-Kisber & Stewart, 2009; Cutts & Waters, 2019) and within phenomenological research (Dufrenne, 1978; Prendergast, 2009; Prendergast et al., 2009) but, to the authors’ best knowledge, has not been used in this way within medical education.

Construction of participant-voiced poetry is compatible with hermeneutic phenomenology, the research approach adopted by this study. Re-portrayal of participant lived experience as poetry is a method of embodied interpretation- “an approach to re-presenting… experiences, rather than using primary autobiographical sources” (Galvin & Todres, 2009) and represents a method of data analysis in and of itself, as writing helps researchers find out more about themselves and their topic of inquiry (Richardson, 2000). In line with interpretative phenomenological tradition, poetic inquiry seeks to represent human experience in all its complexity, encouraging researchers to step beyond their own immediate world, into the accounts of their participants. For many phenomenologists, poetry is essential to phenomenological thought- Van Manen, for example, refers to phenomenological exploration as a ‘poetising project’ (Van Manen, 1997), whilst, in his later life, the phenomenologist Heidegger drew upon poetry to further his philosophical enquiry (Heidegger, 1971). Galvin and Todres (2009) methodological research concerning poetic inquiry is based on the phenomenology of Gendlin and Gadamer- specifically, Gendlin’s concept of ‘felt sense’ and Gadamer’s conceptualisation of understanding and knowledge as a mode of being, as constructed through dwelling amongst the language and poetic conventions of a participants’ account. It is Galvin and Todres’ ‘embodied interpretation’ approach, with its basis in Gendlinalian and Gadamerian hermeneutics that this study aligns itself with philosophically.

According to Galvin and Todres (2009), through the construction of poetry from their participants’ data, researchers attune themselves and their bodies to the language of their data and stories within it, which engenders a ‘bodily felt sense’ regarding the meaning of an account. ‘Felt sense’ was originally described by Gendlin as an ongoing living interaction with the world—it is an internal bodily awareness and sense of meaning about the world that is more than a simple feeling, though emotions may be involved (Gendlin, 1981; Rappaport, 2013). Encountering a rainbow may simply make you feel happy (a feeling), or it may engender a sense of warmth, a flowing and expansive sensation that begins in your chest and radiates throughout your body- this is a felt sense (not the only one, there are many sorts) and is what poetic inquirers must strive for as they make embodied interpretations. The embodied interpretation of felt senses can be difficult to articulate, but the process of poetic inquiry and construction of participant-voiced poems allow researchers the space and depth to find ‘words which work’ (Galvin & Todres, 2009). Simply put, creative work is what most readily flows from the felt sense (Gendlin, 1992). Such an expression of embodied interpretation using poetic language, as Galvin and Todres (2009) so nicely surmise, ‘emphasises ‘wholeness’, in that, through rhythm, repetition, and imagery, a wholeness is pointed to that is more than what is there’. Through the use of participant-voiced poetry, therefore, phenomenological thought is embraced- poetry emphasises language, attunes researchers to the lived experiences of their participants (Richardson, 1992; Rosenblatt, 1978), and facilitates reflexivity (Freeman, 2001). As the use of participant-voiced poetry “can alter the way we understand phenomena” (Eisner, 1991; Sjollema et al., 2012) we hoped its use would reveal new insight, particularly into the “affective experiential domain” (Prendergast, 2009) of participant experiences of transition.

Though participant-voiced poems mobilise and collate the verbatim words of research study participants, they are a construction of the research team- an interpretation of participant experiences, as with to many forms of qualitative data analysis. Within this context, participant-voiced refers to the fact that such poems are constructed through *exploration* of participant experience, in contrast to researcher-voiced poems, which are written from the researcher’s own experiences (often concerning the research process) and literature-voiced poems, poems written by researchers about theory or a literature base (Van Luyn et al. 2016). There has been some debate concerning the appropriateness of the term ‘participant-voiced’ to describe poems which are ultimately researcher constructions (Cahnmann-Taylor and Zhang, 2020). Gadamer (1975) conceptualises understanding within interpretative phenomenology as ‘a *play* between context and detail, the personal and the relational, the past and the future’ (Galvin & Todres, 2009). In line with Gadamer’s reasoning, participant-voiced poems, although researcher authored, can be seen as communicating a shared sort of understanding- the poems constructed reflect a meaning that has been created in confluence *between* two people. They do not solely represent the voice of the participant or the voice of the researcher, they represent the voices of both. Embodied interpretation is the place where the experiences of both parties meet, whilst the uniqueness of individual participants’ experiences are respected by researchers through immersion in their story and respect of the ways in which they employ language.

Practically, all poems were constructed by one author (MB) using participants’ own words, represented in chronological order to exemplify each second-order construct. Once sub-themes had been identified through traditional phenomenological inquiry, second-order construct poems were also connected for each participant to form sub-theme poems where possible. All authors are experienced writers, interested in poetry, although this was the research team’s first use of this method. Efforts were made to attempt to replicate participant rhythm, emphasis and tone, as Butler-Kisber and Stewart recommend (2009), with each interview’s audio recording reviewed in detail, and notes made concerning participant tone, emphasis, use of pauses, rhythm and syntax. Table 2 provides more details on the practical steps within analysis taken by the research team, in line with Ajjawi and Higgs’ 6 steps, and in regard to the construction of participant-voiced poetry.

**Table 2:** How the research team enacted Ajjawi and Higgs’ 6 stages of hermeneutic analysis, including integration of participant-voiced poetry

Ajjawi and Higgs’ analytic stage	Activities in line with Ajjawi and Higgs’ recommendations	Activities using and integrating research poetry
1. Immersion	The research team manually transcribed all data to foster familiarity AP and MB then engaged in repeated readings of the transcripts, both independently identifying first order constructs from all transcripts Following this initial identification, AP and MB met to discuss the constructs, subsequently returning to the data to deepen their own understanding as to the meaning of the previously identified first-order constructs	Each interview transcription and raw audio was reviewed in detail and notes made in the margins of transcripts regarding participant tone, emphasis, use of pauses, rhythm and syntax Transcripts were reviewed for repeating words, phrases and expressions Butler-Kisber recommends researchers make efforts to replicate participant emphasis and tone in prose, this process emphasised these considerations
2. Understanding	MB developed an overall set of first-order constructs, taking into account previous discussions held with AP. Where possible, first-order constructs were expressed using participants’ own words Following creation of one list of first order constructs, the entire research team met to discuss the final list in depth Further to this detailed discussion, GF reviewed three randomly selected transcripts, examining the list of first-order constructs alongside original data for concordance	
3. Abstraction	MB, AP and GF met discuss their personal observations and understandings of first-order concepts Where relevant, and in line with other work utilising this framework (Bynum IV et al., 2019), educational theory was discussed at this stage. Multiple and multidimensional transition theory (MMT) was identified as relevant The entire team formed a list of second-order constructs. MB used this list to re-analyse all 7 transcripts As the second-order constructs were founded in the meaning of multiple individuals, GF secondarily re-analysed all transcripts using the list of second-order transcripts Multiple discussions were held throughout this process in order to deepen analysis and resolve discordance A whole group meeting (MB, AP, NY, GF) was undertaken, where second-order constructs were reviewed, ensuring credibility and concordance with original data Second order constructs were grouped into sub-themes by MB and GF in a joint meeting	The content of each transcript was reviewed to highlight poignant sections of narrative or metaphor relevant to the list of second order constructs identified from traditional analysis MB constructed one participant voiced research poem for each second-order construct identified. Each poem was taken from one participants’ interview, displaying their own words in chronological order in an attempt to represent underlying meaning, whilst maintaining participant voice Within the whole group meeting (MB, AP, NY, GF) participant-voiced research poems were evaluated alongside second-order constructs. Scrutiny of research poetry alongside the agreed list of second-order constructs not only deepened analysis and prompted reflexive discussion, but refined initially presented poetry, ensuring fit
4. Synthesis	Sub-themes were grouped into themes. This was facilitated by returning to data immersion alongside engaging in the contemporary transition literature base Cycles of writing were undertaken by MB to elaborate first upon sub-themes, and subsequently upon identified themes A document including this writing in full and all research poems was circulated to the entire research team, who then met to analyse the identified themes critically, negotiating changes to add clarity and best represent the underlying first- and second-order constructs. Critical analysis of thematic groupings alongside identified constructs and participant-voiced poetry, allowed for clarification of final themes ensured fit	Participant-voiced research poems for each identified second order construct were reviewed to assist with the grouping of sub-themes into themes As part of the cycles of writing, initial research poems were revised, combining second-order construct poems into sub-theme poems for each participant, where several second order constructs were present in one participant transcript. Creation of these poems facilitated thoughts regarding thematic connections As every participant did not speak of every second order construct contributing to a sub-theme, a small minority of poems did not fully encapsulate described sub-themes and were excluded from final data presentation Research poems and cycles of writing were integrated into one document for presentation to the full research team
5. Illumination	MB examined the connections between identified themes and sub-themes, reconstructing these themes into a narrative form of data presentation	Research poems assisted greatly within this stage, already acting as a form of illustrative narrative highlighting connections between themes in their revised form. MB read and re-read all research poems written for each second-order construct, evaluating them alongside their original data and original second-order constructs for concordance and fit as sub-theme poems
6. Integration	Themes were tested and refined through group discussion of the illustrative narrative and final set of research poems, presented also in narrative format, by MB Original themes were scrutinised alongside the thematic narrative and adjustment made, where necessary, to ensure to fit with, and appropriate portrayal of, participant recounts as a group The thematic narrative and poetic narrative were combined, then reviewed by the team as a whole for concordance	Research poems were presented in narrative format by MB Original themes were scrutinised alongside the poetic narrative and adjustment made, where necessary to ensure fit with original data Although some second-order poems were excluded when they could not contribute to a full sub-theme for a participant, use of research poetry alongside traditional analysis ensures all data is accounted for and presented The poetic narrative and thematic narrative were combined, then reviewed by the team as a whole for concordance

## Results

This work’s results are presented thematically, in narrative format, integrating participant-voiced poetry. Participants are given pseudonyms and demographic information provided after each quote. Four overarching themes were identified: the nature of transition to practice; the influence of community; the influence of personal beliefs and values; and the impact of unrealistic undergraduate experience.

### The nature of transition to practice

For some participants, the transition from student to physician brought unanticipated enjoyment.

I never thought I would enjoy [it] so much, I wasn’t expecting that so much. Eric, M, F2

Most participants’ experiences, however, lived up to the reputation of transition as a stressful time. Even for those who found unanticipated joy in practice, early experiences demonstrating the weight of their new medical responsibility proved stressful.

I’d wake up in the night having on-call dreams… it was stressful. Eric, M, F2

I found it immensely stressful. One day I was a student, the next a doctor with real people depending on me to make real decisions… that could harm someone. Liam, M, F1

Most participants, as may be anticipated, experienced both joy and stress in their work. Any dichotomy created between these two states would be a false one. Yet, in sum, descriptions describing stressful, unenjoyable experiences were more prominent. Some contextualised transition to FY1 as the most stressful, or largest step of their life.

It’s been difficult… challenging… I think the step from medical school to becoming a doctor has been the biggest learning curve I’ve ever experienced in my life. Imogen, F, F1

Stress during transition was multifactorial but of particular prominence was the influence of fragmented postgraduate training schemes. Short job rotations of four months made forming meaningful relationships difficult.

During F1…it’s so hard to cultivate that sense of belonging when you’re only in one place for four months. Emma, F, F4

I don’t think that the team around you ever fully invests in you because you’re just temporary… Claire, F, F3

Workforce pressures within the NHS detracted from trainee support and increased workload, creating arduous working conditions for trainees.

It’s chronic the understaffing… rota gaps are everywhere… you’re often taken from your ward to cover another one where you have no idea what’s going on or who to turn to for support or have to do that alongside all your regular work… It’s like fighting a losing battle…you’re bound to be injured. Liam, M, F1

Such pressures contributed to a sense of ‘being thrown in the deep end’ and expectation of trainee autonomy beyond that for which they felt prepared. Responsibility was most clearly highlighted during out-of-hours work.

I was thrown in the deep end quite seriously in F1… my first day, I was pretty much the only one [there]… Eric, M, F2

That sort of awakening moment when you have to tell yourself it’s you there, you’ve got the responsibility…you’ve got patients’ lives literally in your hands…those experiences were often on-calls… Emma, F, F4

A sense of competence was key to feelings of belonging as a doctor. Traumatic clinical experiences, such as the unanticipated death of a patient or implication in a medical error were triggers for self-doubt regarding competency. Such situations were borne of workload pressures or understaffing leading to inadequate support. Adequate debriefing of trainees following such events was not present, leading to long-term rumination of competence.

A few weeks in I prescribed the wrong dose of warfarin to a patient out-of-hours… it’s one of those jobs that shouldn’t even be done on-call…but everyone during the day is so swamped… thankfully the nurse picked up it was a big increase in dose and rang me to double-check… I think I cried every night that week after that… and the days at work I was anxious and slow…. I doubted myself for a long time after that…I still do. Liam, M, F1

I did think there would be more understanding from my supervisor… but they were very accusatory… it was more, this is a progression concern, you’re incompetent… rather than trying to understand why it happened… if your consultant tells you you’re not competent…you don’t feel competent.” Liam, M, F1

In opposition to this, trainees identified positive senior feedback regarding autonomous management of acute situations to be validating. External validation of competence generated a sense of ‘being a proper doctor’.

I had an on-call… somebody who’d taken a bit of a turn for the worse…you go, “Oh I think this is what’s happening. I’ll do this, this, this and this.” And then you call the reg and the reg goes, “Yeah, that’s fine.” You know? You got everything. And then you’re like, “Okay, cool.” So my brain does function, it got all that. It’s well enough to do this job. I think that was the first time I felt like a proper doctor. Eric, M, F2

**Fig. 1:**
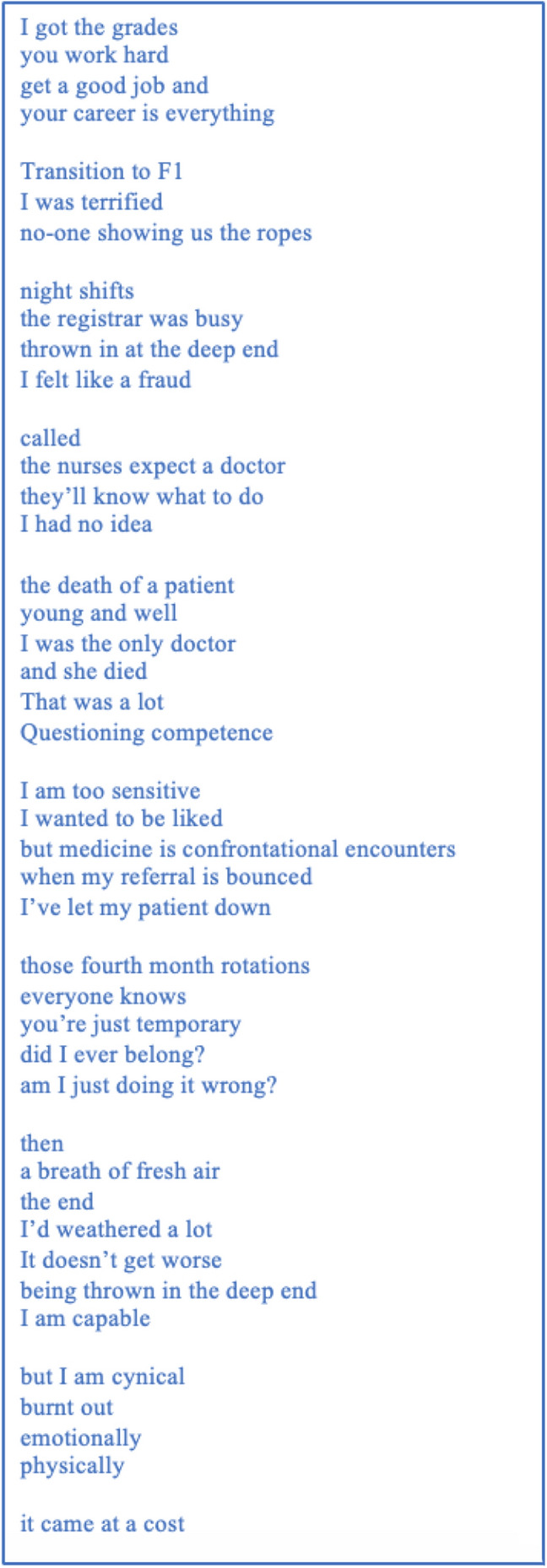
“It came at a cost”: A poem on the sub-theme ‘Factors contributing to burnout’

For some, the above stresses cumulated in feelings of burnout. A participant-voiced research poem written from Claire’s transcript (Fig. 1) emotionally elaborated the lived experience of the stress involved in transition to practice, and how such stress can escalate to burnout, despite development of a sense of competence. This poem, entitled ‘It came at a cost’ clearly demonstrates this connection.

### The influence of community

Newly qualified doctors commented on the influence of a variety of communities they were now a part of through working as a doctor. Camaraderie with a variety of multidisciplinary team members fostered belonging. Simple actions, such as making an effort to call new doctors by their names, and taking an interest in them as people, helped new doctors feel like more established members of the community.

I’ve gotten on really well with everyone and that’s what’s kept me going when work’s awful. You’ve got great nurses behind you…you get on well with the physio, or dietitian... Eric, M, F2

Simple things like knowing your name and it sounds really silly…pathetic, but just when you’re in a ward round. For the consultant to say, “Oh, [Thea] will do that for you,” not, “We’ll get one of our F1s to do that”. Thea, F, F2

However, not all participants experienced belonging to the community in which they worked. Frequent rotations and professional conflict left trainees feeling unsupported.

There are a lot of times in medicine where you have difficult conversations with colleagues…the person that you're referring to doesn't want to take [your] referral or when…people are trying to give you work to do and you have to explain that you haven't been able to do it [yet]… It can be quite confrontational….and because I'm quite friendly…I really struggle… Claire, F, F3

It’s almost impossible to put down any roots… you get to know people, then have to move again and start over. Liam, M, F1

**Fig. 2:**
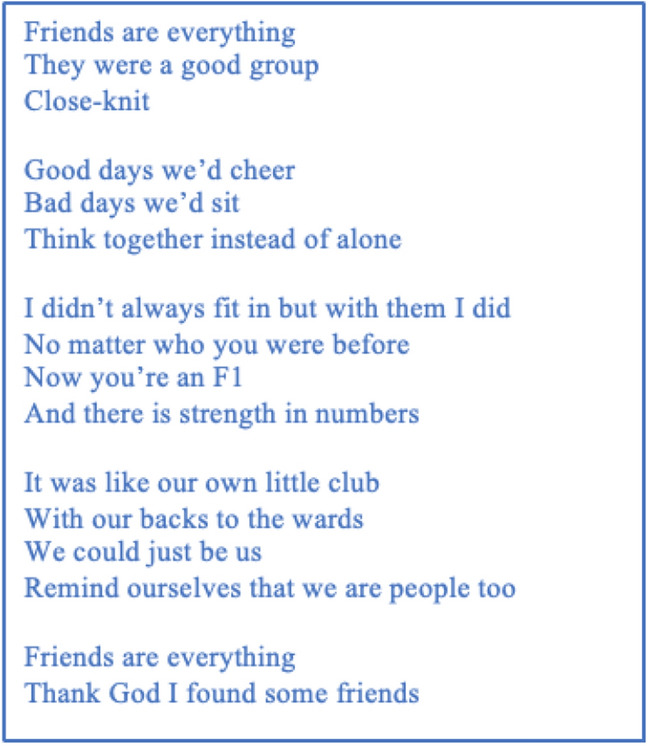
“Friends are everything”: A poem on the sub-theme ‘peer support and belonging’

Participants voiced positive experiences of support found amongst their peers. A participant-voiced poem, written from Liam’s transcript on the sub-theme ‘peer support and belonging’ portrays one lived experience of a community of FY1 peers (Fig. 2).

### The influence of personal beliefs and values

**Fig. 3:**
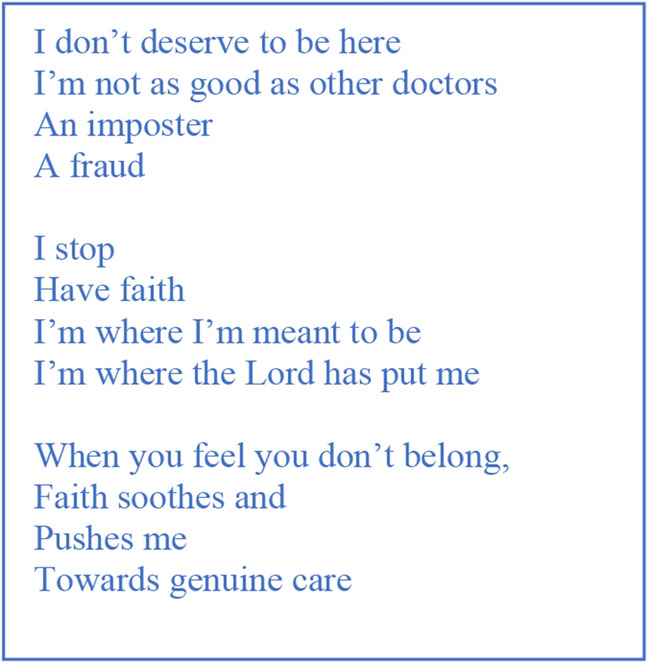
“Where I’m meant to be”: A poem on the sub-theme “The influence of beliefs on affirmations of purpose”

When discussing transition to practice, newly qualified doctors often spoke of possessing a strong set of personal beliefs and values which acted as guiding lights in times of hardship. A participant-voiced research poem is written from Eric’s transcript (Fig. 3, below) encapsulating this guidance in moments of difficulty.

Participants with family members in medicine voiced being guided by knowledge of the ‘reality’ of medicine, and the role this played in the development of their own beliefs.

I come from a family of doctors… I’ve always seen what it’s like with my dad… he always told me you’ve got to pay it back, the privilege we have…it’s definitely influenced how I practice. Liam, M, F1

Most often, participants viewed their purpose as being one of care. In a professional context, this manifests through ensuring patient-centered care.

I think I try and treat my patients as…humans and more holistically… [that] helps me in those moments where I wonder what I’m doing. Emma, F, F4

Creating meaningful connections with patients led to many participants’ first instances of feeling like a doctor.

I always thought it would be the things like, “Oh, you’re going to a cardiac arrest and you get ROSC [Return of Spontaneous Circulation],” and that’s gonna be what makes you feel like a doctor but it’s not that at all for me. It’s when you’re speaking to a patient… and you make them feel better through that…it’s not the thing that I expected to get the most achievement out of. I thought it would be the drugs you prescribe, the intervention[s]… it’s more just when you are actually just sitting, communicating with patients …that’s when you feel like a doctor. Imogen, F, F1

**Fig. 4:**
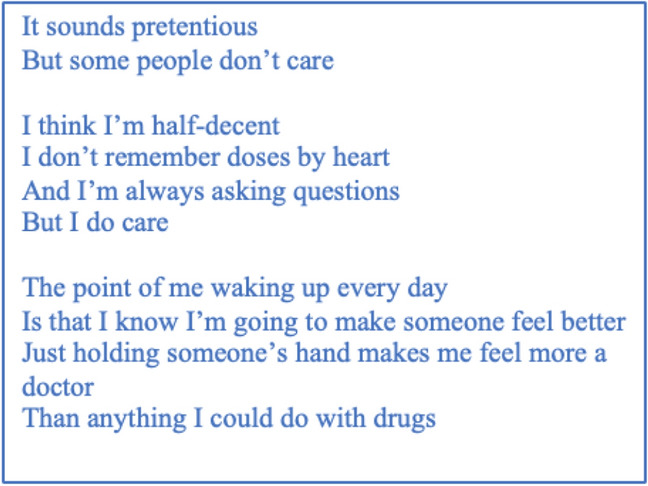
“Just holding someone’s hand”: A poem on the subtheme “The influence of an ethic of caring”

A participant-voiced poem (Fig. 4) is written from Thea’s transcript, encapsulating this ethic of caring in action- as clinical experiences align with personal values, meaningful patient connections increase feelings of ‘being a doctor’.

When environmental pressures, such as time pressures, detracted from the ability to offer patient-centered care, trainees suffered distress, forced to practice in a manner incongruent with their personal values. For some, this distress culminated in feelings of burn-out.

It was very difficult… rushing…not having the time to give the care [patients] need… that I want to offer…that was a point of conflict for me. Liam, M, F1

It has been very different than what I thought it would be… it has upset me when I have to rush patients…they deserve better… and [that has ] started to wear me down… I worry it’s burning me out. Charlotte, F, F1

### The impact of unrealistic undergraduate experience

Participants noted that learning how to be a doctor is something that takes place within a clinical environment. There were suggestions that undergraduate study and examinations did not represent the realities of practice.

I don’t think I actually learnt how to be a doctor in medical school… that teaches you one thing… and then the wards teach another. Liam, M, F1

The transition was difficult in terms of how much can I do as an F1 and what exactly is my job? Because you go through school and they teach you about…how you deal with heart attacks and epilepsy… when you’re in a team of people… realistically, you’re just taking the blood or writing the drug chart. So, that’s really weird going from having to know everything at the finals to… being a little cog in the machine…someone else is making decisions you thought you had to make. Thea, F, F2

Shadowing as a student, even during the assistantship period, was seen as largely passive, in contrast to the role of a doctor, which was perceived as assertive.

A lot of places you just kind of feel like a piece of furniture…. Imogen, F, F1

As a doctor you’re expected to… [have] authority, but as a student it was mostly just watching and trying not to get in the way. Liam, M, F1

Although most doctors felt prepared regarding clinical knowledge for transition to practice, most felt ill-prepared to deal with the responsibility of being a foundation doctor.

I don't think you can [prepare] until you actually start working… because… you’ve not got any responsibility until you actually start … Charlotte, F, F1

Given the disingenuousness of undergraduate education, FY1 was the site of many transformative experiences. Several transformational shifts were volunteered by participants including regarding clinician responsibility, dealing with uncertainty, and understanding that helping doesn’t always mean fixing. These transformational shifts were often troublesome and emotional. As they are experienced alongside other previously described and frequently encountered stressors in FY1, transformative experiences can trigger trainee distress and necessitate additional support as trainees come to terms with shifts in how they view the world. Despite the difficulties transformative experiences may bring, once processed, they change trainee outlook significantly. Trainees perceived transformative change as helpful for future practice.

**Fig. 5:**
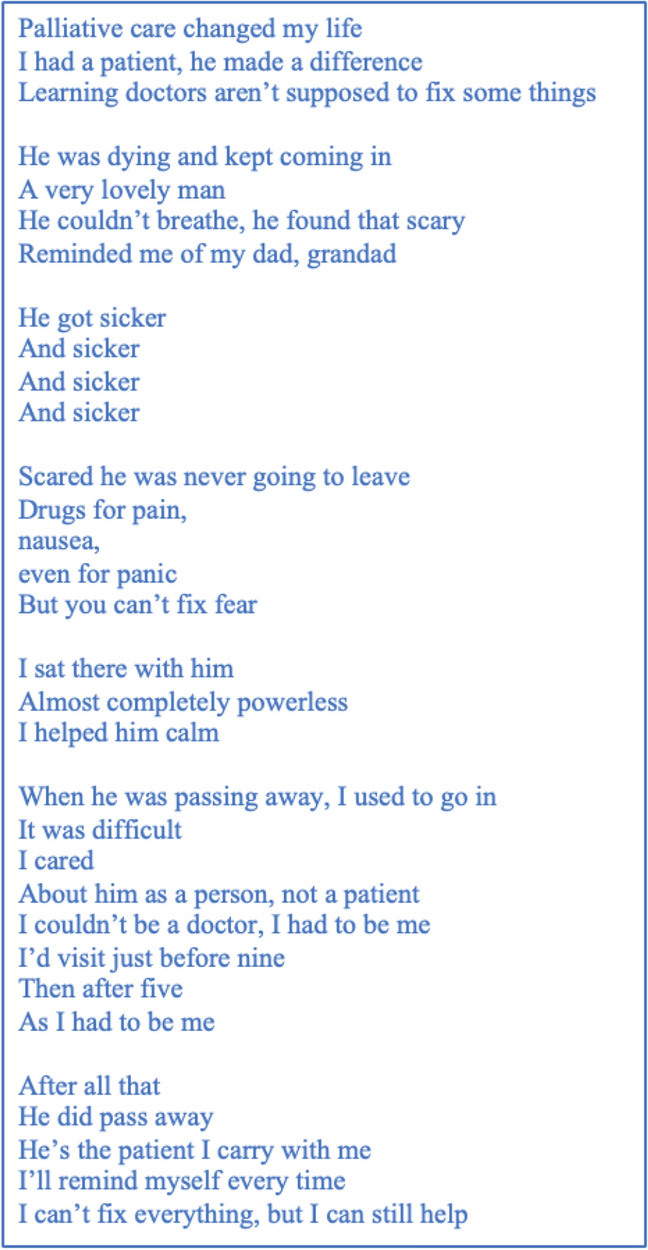
“Doctors aren’t supposed to fix some things”: A poem on the sub-theme ‘The influence of transformational clinical experiences on participant understanding of the concept of ‘help’

Thea’s transcript was used to create a participant-voiced poem (Fig. 5) regarding the process and influence of a transformational clinical experience.

It is important to note that, although Thea describes an ultimately positive transformation in outlook, not all experiences were well-processed by participants. Some trainees remained stuck in a state of liminality, where they struggled to integrate new clinical experiences into their pre-existing schemes of knowledge, beliefs and values. This proved distressing, as trainees experienced unresolved conflict between their practice as a new doctor and the way in which they see, and often wish to keep seeing, the world.

… and everyone kept telling me it was something I just needed to come to terms with… medicine is all about not knowing… I am such a sure person normally; I didn’t want to accept that we couldn’t do more to find out [what was happening] for [that patient]… I find it challenging…I don’t think my supervisors get it… Charlotte, F, F1.

## Discussion

As newly transformed butterflies learn the lay of the land to help them survive, so must new doctors. Yet, every four months, new doctors are uprooted, forcibly transported to a new physical, social and cultural environment like a butterfly ensnared by a net. Such frequent rotation means foundation training lacks continuity, an attribute of training programmes that fosters interpersonal connection (Hirsh et al., 2007). Interpersonal relationships play an important role in transition- previous research utilising MMT theory highlights interpersonal connectivity as a factor facilitating successful transition, particularly within the ‘social domain’(Gordon et al., 2017). Yet, interpersonal relationships during the foundation programme are difficult to establish (Magola et al., 2018; Wells et al., 2019). Although new doctors experience longitudinal continuity of supervision (‘care… over time, but without an ongoing relationship’ (Boulton et al., 2006)) through their assigned supervisors, they experience little-to-no interpersonal continuity, where relationships involve trust and meaning (Haggerty et al., 2003). Poor educational continuity, therefore, is a barrier to forming interpersonal relationships within MTT’s social domain that facilitates successful multiple transitions as a new doctor. It is likely restructuring of the foundation programme is necessary to pay better heed to fostering interpersonal relationships. The Northwest of England Foundation School piloted Longitudinal Integrated Foundation Training (LIFT) in 2016 (Burnett et al., 2018), within which foundation doctors spend 1.5 days/week in the same general practice for their 2 years of training. Early positive benefits have been reported, including more effective supervisory relationships, emphasising pastoral support (Burnett et al., 2018). Increased provision of LIFT schemes nationally could begin to tackle the deficiency of continuity observed within this research, improving support whilst balancing the need for a degree of clinical challenge and change (Gill, 2013; Williams & Ledger, 2019).

Further to the above, this work demonstrates the importance trainees place in the values that guide their actions- a psychological domain within which transition occurs in MMT. The value most frequently spoken of by participants within this work was an *‘ethic of caring’*, empathic beliefs participants possess that are rooted in notions of care and direct decision-making. Noddings (1988) was one of the first to describe this ethic as a feminist approach to morality (Balmer et al., 2016). Developing an ethic of caring has since been referenced in work evaluating Longitudinal Integrated Clerkships (LICs) as the orientating perspective students assume as they become increasingly patient-centered (Konkin & Suddards, 2012). This work builds on these findings, demonstrating an ethic of caring as a fundamental element of the psychological MMT domain guiding new doctors’ through transition. When previously described, this ethic is framed positively, as improving student relationships with patients (Konkin & Suddards, 2012). Our work echoes this, but casts new light as to how adoption of this very necessary ethic within the current fraught landscape of the NHS predisposes trainees to moral injury and burnout. Work within undergraduate education suggests ‘values-challenging’ educational environments cause ‘erosion of empathy…and burnout’ (Gaufberg et al., 2017; Brazeau et al., 2010; Christakis & Feudtner, 1997; Dyrbye et al., 2008; Feudtner et al., 1994; Haidet et al., 2002; D. Hirsh, 2014; Hojat et al., 2009). This work supports these findings. As new doctors transition to practice, they come to work in an occupational environment at odds with one of their core values, their ethic of caring. As a consequence, moral injury and burnout can ensue. Recent focus on the notion of personal resilience within medicine misses the mark in its attempts to lessen burnout (Meeks et al., 2019). Moral injury here is rooted in systemic health service issues, so solutions to this epidemic must also be systemic in nature (Taylor, 2019). We must do better to promote a healthcare system rooted in values of care, with adequate funding to realise these values. To the author’s best knowledge, this is the first work providing a weight of empirical evidence to the notion of moral injury during transition to practice, and the first work highlighting a connection between trainees’ ethics of caring and moral injury.

### Reflections on the use of participant-voiced poetry

Although the central focus of this paper is a phenomenological exploration of new doctors’ transition to practice, as this is the first use of participant-voiced poetry in medical education research, we felt it necessary to briefly reflect on the impact of this method.

In several instances, poetry construction altered the way in which transition to practice was understood by the research team. Although key themes were evident from traditional phenomenological inquiry in a typically subdivided fashion, the creation of research poems allowed for more considered reflection on how stories were told, and the connections between themes. For example, the creation of the poem ‘It came at a cost’ more clearly highlighted the emotional impact of transition within a fragmented system. As Claire spoke of her difficult transition to practice, she was clearly emotional, and her narrative became fragmented. Paying heed to how this communicated Claire’s experience through creation of participant-voiced poetry revealed the emotional impact of transition akin to moral injury, as her fragmented narrative paralleled the fragmented state of new doctors’ training. Integrating participant-voiced poetry into phenomenological inquiry has not only cast light on the affective experience of transition but has illuminated the experience of a complex phenomenon in depth- moral injury. Through detailed interpretation of what was said, how things were said, and what was left unsaid, participant-voiced poetry helped the research team step into participants’ experiences and reflexively consider their own interpretations of participants’ narratives as they constructed the poems. We anticipate the method of participant-voiced poetry could align with a variety of qualitative research questions but would be of particular use for those conducting in-depth, phenomenological studies.

### Limitations

As transition to practice occurs in FY1, it could be argued for a homogenous sample FY1s alone should have been recruited. Although we acknowledge this stance, we purposively chose to expand sampling to increase recruitment. In phenomenological research, homogeneity refers to a ‘probable shared perspective on a phenomenon of interest’ (Larkin et al., 2019). All participants had experienced transition to practice at the time of this research. Given this, and the fact interview questions were designed to prompt reflection upon transition, this sample can be viewed as largely homogenous. That said, future in-depth research with more homogenous groups of participations exploring transition to practice would add depth and nuance to these findings. It is also important to note this work evaluated initial transition to practice within a 2-year programme. Although this has generated detailed discussion regarding initial transition, examination of different stages of the FY programme may yield different results. Future work should focus on detailed longitudinal investigation of the foundation programme.

Although our in-depth analysis of new doctors’ lived experience of transition to practice has cast light on moral injury occurring within psychological, social and physical domains of multiple and multidimensional transitions theory, the influence of transition within MMT’s cultural domain on moral injury was less evident within the data of this study. Future research questions should investigate moral injury specifically through the lens of MMT’s cultural domain, to add to this broad phenomenological conceptualisation.

As this research was conducted within the UK, context must be considered when transferring findings. The UK NHS is free at the point of delivery, and so issues regarding resourcing and funding treatment at an individual patient level are less prevalent than in countries with private healthcare systems. We postulate doctors’ ethics of caring, which this research found to play a role in moral injury, may be challenged even further within systems where patients cannot afford to pay for care. Further research is necessary to translate the findings of this work, which detail challenges to doctors’ ethics of caring within an underfunded public system, to other healthcare settings which may challenge this ethic in different ways.

## Conclusion

Robert Frost is quoted as having said ‘writing a poem is discovering’ (Thompson, 1959) and, in the case of this research, this rings true. Incorporating participant-voiced poetry into hermeneutic phenomenology has cast new light on how doctors experience transition to practice. Of particular note are the difficulties postgraduate trainees face as they work within a fragmented training system, devoid of educational and supervisory continuity. All new doctors described an ethic of caring as a core value governing their transition. However, possession of this ethic in the fraught landscape of the NHS is a double-edged sword, predisposing new doctors to moral injury as they work in an underfunded system misaligned with their ideals. This work contributes to existing knowledge regarding new doctors’ transition to practice and adds depth to early findings regarding the importance of postgraduate continuity and the presence and cause of clinician moral injury. Educational authorities should consider revision of the foundation programme to increase interpersonal continuity, so improving trainee support during such a turbulent time. Focus on individual doctors’ resilience should be redirected to tackle larger, systemic health service issues, as improving working conditions is likely to decrease the risk of moral injury. Like butterflies- nay, most creatures—new doctors thrive given continuity, care, and a role that allows them to flourish symbiotically within the ecosystem of clinical practice. Yet, current training within the UK quickly clips new doctors’ wings. Change is urgently needed to combat the rising epidemic of moral injury that training is, in part, responsible for.

## Author contributors

MELB and GMF had the original idea for the research. MELB, GMF and AP created all study documentation and sought relevant ethical approval. MELB and AP recruited all participants and undertook all interviews and transcription. All authors were all substantially involved in data analysis. MELB prepared the initial draft of this paper, which was sent to all authors for their comments and edits. All authors approved the final version of this manuscript prior to submission.
